# A multi-bin rarefying method for evaluating alpha diversities in TCR sequencing data

**DOI:** 10.1093/bioinformatics/btae431

**Published:** 2024-07-01

**Authors:** Mo Li, Xing Hua, Shuai Li, Michael C Wu, Ni Zhao

**Affiliations:** Department of Mathematics, University of Louisiana at Lafayette, Lafayette, LA, 70504, United States; Public Health Sciences Division, Fred Hutchinson Cancer Center, Seattle, WA, 98109, United States; Department of Biostatistics, Johns Hopkins University, Baltimore, MD, 21205, United States; Public Health Sciences Division, Fred Hutchinson Cancer Center, Seattle, WA, 98109, United States; Department of Biostatistics, Johns Hopkins University, Baltimore, MD, 21205, United States

## Abstract

**Motivation:**

T cell receptors (TCRs) constitute a major component of our adaptive immune system, governing the recognition and response to internal and external antigens. Studying the TCR diversity via sequencing technology is critical for a deeper understanding of immune dynamics. However, library sizes differ substantially across samples, hindering the accurate estimation/comparisons of alpha diversities. To address this, researchers frequently use an overall rarefying approach in which all samples are sub-sampled to an even depth. Despite its pervasive application, its efficacy has never been rigorously assessed.

**Results:**

In this paper, we develop an innovative “multi-bin” rarefying approach that partitions samples into multiple bins according to their library sizes, conducts rarefying within each bin for alpha diversity calculations, and performs meta-analysis across bins. Extensive simulations using real-world data highlight the inadequacy of the overall rarefying approach in controlling the confounding effect of library size. Our method proves robust in addressing library size confounding, outperforming competing normalization strategies by achieving better-controlled type-I error rates and enhanced statistical power in association tests.

**Availability and implementation:**

The code is available at https://github.com/mli171/MultibinAlpha. The datasets are freely available at https://doi.org/10.21417/B7001Z and https://doi.org/10.21417/AR2019NC.

## 1 Introduction

Human T cells, originating from hematopoietic stem cells, recognize foreign antigens through the highly diverse T cell receptor (TCR) on their cell surface (Joyce 2001). TCR diversity is achieved through V(D)J recombination, randomly combining gene segments encoding *α* and *β* chains (or *γ* and *δ* chains) (Kappler *et al.* 1987, Davis and Bjorkman 1988). This process, along with the chain pairing, results in over 1e18 possible recombinations (Petteri Arstila *et al.* 1999). Diverse TCR repertoires are crucial for antigen recognition, immune response versatility, immunological memory establishment, and autoimmune reaction prevention, unveiling significant clinical and biological distinctions among individuals.

Alpha diversity, a within-sample diversity measure assessing the richness and/or evenness of TCR repertoires, is frequently used in the literature to characterize immune responses to disease progression or changes in disease status (Lang Kuhs *et al.* 2018). It has also been used to detect phenotype differences between healthy individuals and those affected by diseases (Robins *et al.* 2009, Emerson *et al.* 2017, Azizi *et al.* 2018). However, alpha diversity is influenced by the sequencing intensity (namely, “library size,” “sequencing depth,” or “total reads”) (Willis 2019, Colbert *et al.* 2022, Hong *et al.* 2022). Larger library size generally corresponds to stronger sequencing effort and thus, higher alpha diversity. This is a technical artifact from sequencing rather than a true biological signal. Therefore, normalization of alpha diversity measurements is essential to account for the variation in library sizes across samples (Lande 1996). Normalization aims to mitigate technical biases from differential library size, ensuring that observed diversity patterns accurately represent the underlying biological associations of interest.

Rarefying is the most widely used approach for normalization in alpha diversity analysis. First proposed by Sanders (1968), the gist of rarefying lies in subsampling the sequencing reads of all samples to the same level (often the lowest level across all samples after quality control), ensuring equal numbers of remaining reads for comparison (Hong *et al.* 2022). It has been extensively used to compare alpha or beta diversities between clinical conditions in microbiome studies (Xia *et al.* 2018) and BCR/TCR sequencing studies (Venturi *et al.* 2007, Laydon *et al.* 2015, Rosati *et al.* 2017, Bortone *et al.* 2021). Despite its popularity, the overall rarefying approach is controversial as it discards sequencing reads, potentially increasing measurement error and losing statistical power (McMurdie and Holmes 2014, Hong *et al.* 2022). It also introduces variation through reliance on a single rarefying replicate (Cameron *et al.* 2021, Hu and Satten 2022). Concerns about its validity have been raised, as it may introduce bias when addressing underestimated alpha diversity due to under-sampling (Willis 2019). It has also been criticized by Hurlbert (1971) and Simberloff (1972) for its tendency to overestimate the expected number of species. Finally, positive correlations have been found between the total reads and the alpha diversities calculated from the rarefied samples (Lang Kuhs *et al.* 2018, Colbert *et al.* 2022), indicating that rarefying does not fully remove the dependency of alpha diversity estimates on sequencing depth.

Instead of an overall rarefying approach, we propose a novel “multi-bin” rarefying method to mitigate the confounding effect of library sizes, stratifying the data into multiple subsets based on library sizes. By rarefying within subsets, we adjust the rarefying levels of TCR repertoires with larger library sizes to higher levels and those with smaller library sizes to lower levels. This has the advantage of retaining all samples while significantly minimizing the loss of sequence reads. We show via simulations that our approach can effectively reduce the potential dependence between library sizes and alpha diversity, better control the type-I errors, and improve the statistical power in association tests compared to alternative strategies. The approach is applied to a real study examining the association between TCR profiles with cytomegalovirus (CMV) exposure.

## 2 Materials and methods

The central observation is that overall, rarefying fails to decrease dependency on read depth, possibly due to nonlinear effects, while rarefying within a more narrow, local range can remove the dependency. Thus, the overall objective is to use a “multi-bin” rarefying method for accommodating varying library sizes, wherein we bin samples based on sequence depth, conduct analyses within bins, and then meta-analyze across the different bins. Finally, we describe a TCR sequencing dataset that will be used for simulations and serve as a real data example.

### 2.1 Dividing samples based on read depth

Suppose we have a total of *N* samples (which pass the quality control criteria). Let *L_i_*, i=1,…,N, be the library size of the *i*th sample. We will classify all samples into *K* different bins based on their library sizes, with the *k*th bin denoted by *M_k_* with *n_k_* samples. In specific, let $\left\{c_{1},\ldots ,c_{K-1}\right\}$ is a set of *K—*1 pre-determined threshold parameters satisfying

$$\min(L_{i})=c_{0}\leq c_{1}<\ldots <c_{K-1}\leq c_{K}=\max(L_{i}).$$

Then, sample *i* will be classified into *M_k_* if $c_{k-1}\leq L_{i}<c_{k}$.

Choices of the threshold values ($\left\{c_{1},\ldots ,c_{K-1}\right\}$) should depend on the distribution of all *L_i_*’s. Although these cutoffs are user-defined, some rules of thumb include (i) the range of each *M_k_* should not be too extensive to ensure homogeneity of samples within each bin, (ii) each *M_k_* should have an adequate number of samples, and (iii) there should be no or little correlation between alpha diversities and library sizes within each bin. Adhering to these principles will mitigate the confounding influence of library sizes, offer better type I error control, and reduce the information loss from rarefying.

After each sample is binned, the samples in bin *M_k_*, where k=1,…,K, have library sizes between $c_{k-1}$ and *c_k_*. Within *M_k_*, we set the rarefying level $L_{k}^{*}=c_{k-1}$, rarefy (i.e. subsample sequences without replacement) all samples to $L_{k}^{*}$ (the lower bound of *M_k_*), and calculate alpha diversity correspondingly. Compared to the overall rarefying approach, our “multi-bin” strategy achieves a better balance between the number of samples and the depth of sequencing reads, thereby preserving more information. Within each bin, multiple rarefying can also be used to reduce the impact of random variations introduced by subsampling, with the alpha diversities estimated as the average of multiple rarefying practices.

After obtaining the final alpha diversity measures within each bin, we can conduct the association analysis between the estimated alpha diversities and clinical covariates within each bin. For example, we could use a t-test to compare diversity between two groups or use a Cox model to regress survival on the alpha diversity, each time restricting the analysis to the data within each bin.

At this point, we have *K* separate test statistics, standard errors, and *P*-values, summarizing the association between alpha diversity and the outcome within each of the *K* bins. The final step of our method aggregates the association signals across the *K* bins.

### 2.2 Meta-analysis for aggregating across bins

For each bin *k* (k=1,…,K), let ${\hat{\tau }}_{k}$ represent the effect size estimate of the alpha diversity difference between phenotypes and ${\hat{V}}_{k}$ denote its corresponding variance, which is derived from appropriate models such as *t*-tests or regression models. We adopt the fixed effect meta-analysis model which assumes that all bins have the same “true” underlying effect size *τ* and any observed differences between ${\hat{\tau }}_{k}$’s are attributable to sampling error.

Within the fixed effect meta-analysis framework (Koh *et al.* 2021), the pooled effect (*τ*) across bins can be estimated as a weighted average of every ${\hat{\tau }}_{k}$,

$$\hat{\tau }=\frac{\sum_{k=1}^{K}{\hat{\omega }}_{k}{\hat{\tau }}_{k}}{\sum_{k=1}^{K}{\hat{\omega }}_{k}},$$

where $\left\{{\hat{\omega }}_{k}\right\}$ are the bin-specific weights. The association between the alpha diversity and the phenotype variable of interest could be concluded by testing the null hypothesis of $H_{0}:\tau =0$ against the alternative of $H_{a}:\tau \neq 0$. Particularly, we can assess the significance by a pooled Wald test statistic,

$$W=\frac{{\left(\sum_{k=1}^{K}\omega_{k}\tau_{k}\right)}^{2}}{\sum_{k=1}^{K}\omega_{k}^{2}V_{k}},$$

which, under the null, should follow a $\chi^{2}$ distribution with one degree of freedom (Chen *et al.* 2015).

Multiple choices of weights are possible in this fixed-effect meta-analysis framework. If we assume that the estimates from all bins have homogeneous variances, then using the equal weights ($\omega_{1}=\cdots =\omega_{K}=1$) is a natural choice. This way, $\hat{\tau }$ is simply the average of all ${\hat{\tau }}_{k}$’s. We call this approach the Multi-bin-Equal. If the homogeneous variance assumption is challenged (which is the usual case), such as when the number of samples in each bin differs, it becomes crucial to consider potential factors influencing the heterogeneity. The method proposed by Schmidt and Hunter (2014) involves weighting each ${\hat{\tau }}_{k}$ by the sample size of each bin (*n_k_*) that $\omega_{k}=n_{k}$ for all *k*. In the calculation of $\hat{\tau }$, larger bins with more samples carry more weight than smaller bins with fewer samples. We call this approach Multi-bin-SSW (sample size weighting). Another common approach is the inverse variance weighting, where $\omega_{k}=1/V_{k}$ for bin *k*. The ${\hat{\tau }}_{k}$ is weighted more if bin *k* has a smaller within-bin variance. Under the assumptions of equal underlying effect and homogeneous variability, the inverse-variance weighting strategy is optimal for power (Hedges and Olkin 2014). This is denoted as Multi-bin-IVW (inverse variance weighting). The “multi-bin” approach with these three weighting methods was thoroughly evaluated in the following section through simulation studies.

### 2.3 TCR sequencing data for CMV

To validate our proposed approach, we will extensively utilize the data from Emerson *et al.* (2017) to conduct simulations and assess real data performance. This study aims to identify pathogen exposure signatures from shared TCR sequences. To that end, the T-cell repertoire of 786 healthy bone marrow donors with known CMV serostatus was profiled by immunosequencing. Further details may be found in the original manuscript. Data were downloaded from the immuneACCESS project with access ID (https://doi.org/10.21417/B7001Z). For our purposes, we restricted attention to the data on the 666 samples from Cohort 1.

The data defines a unique TCR *β* sequence as a unique combination of a V gene, a CDR3 amino acid sequence, and a J gene. On average, 242 462 (±102,356 SD) unique TCR *β* sequences are observed on each subject, and the sequencing depth across subjects ranged from 26 648 to 44 119 195. Distributions of sequencing depth for all subjects are plotted in Supplementary Fig. S1. Three of 666 samples have sequence depths <1e5. Additional metadata includes age, gender, inferred CMV status [obtained from the binary phenotype classifier developed by Emerson *et al.* (2017)], and observed CMV status. Supplementary Table S1 provides an overview of these variables.

## 3 Results

### 3.1 Simulation studies

TCR diversity is typically assessed through measures of richness, evenness, or a combination of both within the repertoire. We consider four alpha diversity measurements: unique sequence counts (which takes no account of the frequency of sequences), bias-corrected Chao 1, Shannon index, and Pielou’s evenness. Two simulation studies were conducted to assess the proposed “multi-bin” rarefying approach using the CMV dataset, comparing with various normalization methods listed in Table 1. Linear regression was used to investigate associations between the normalized alpha diversity and variables of interest, treating alpha diversity as the outcome variable. We present the results for unique sequence counts and Shannon index in the main manuscript, while the results for bias-corrected Chao1 and Pielou’s evenness are presented in supplemental files.

**Table 1.** Normalization methods used in simulation studies.

Methods	Descriptions
No rarefying	Alpha diversities are calculated using nonrarefied samples without any normalization.
Overall rarefying	A value $L^{*}>\text{min}(L_{i})$ is selected that all samples with library size lower than $L^{*}$ are discarded, and the remaining samples were subsampled without replacement to achieve the same level of total sequence reads.
LOESS	A LOESS curve with a span parameter of 0.5 is fitted to the estimated alpha diversity from nonrarefied samples, using total reads as the predictor. Model residuals are used in downstream association tests to examine alpha diversity differences between groups.
Multi-bin-Equal	“Multi-bin” approach with equal weighting.
Multi-bin-SSW	“Multi-bin” approach with sample size weighting (SSW).
Multi-bin-IVW	“Multi-bin” approach with inverse variance weighting (IVW).

#### 3.1.1 Simulation A

Our first simulation study focused on evaluating type I errors under conditions where TCR samples exhibit no differences in alpha diversities between phenotypes. We used the entire sequence read data from *n *=* *666 samples in the CMV dataset, randomly designating 333 as cases and 333 as controls.

While sample labels were generated independently of TCR sequences, stochastic variation in each data realization can introduce correlations between library sizes and sample labels. Supplementary Figure S2 displays the histogram of Spearman correlations (*ρ*) between library size and sample phenotype across 100 000 simulations. As expected, the *ρ* values center around zero across all simulations, indicating no systematic confounding between library size and the alpha diversity–phenotype relationship. However, in specific simulations, |ρ| values approached 0.15, suggesting potential confounding effects in those scenarios that could lead to inflated type I errors.

For better illustration, we assessed type I errors across all simulated datasets (|ρ|>0) and subsets with increasing |ρ| thresholds (|ρ|>0.01,…,|ρ|>0.1). All methods in Table 1 were applied to these datasets. In the overall rarefying approach, we set a threshold of $L^{*}=1e6$, and excluded 42 samples with library sizes below $L^{*}$ from further analysis. In the “multi-bin” methods, five cut points (1e6,2e6,4e6,8e6,1e7) were used to create *K *=* *6 bins. Further details on threshold selection can be found in the real data analysis section.

**Figure 1.**
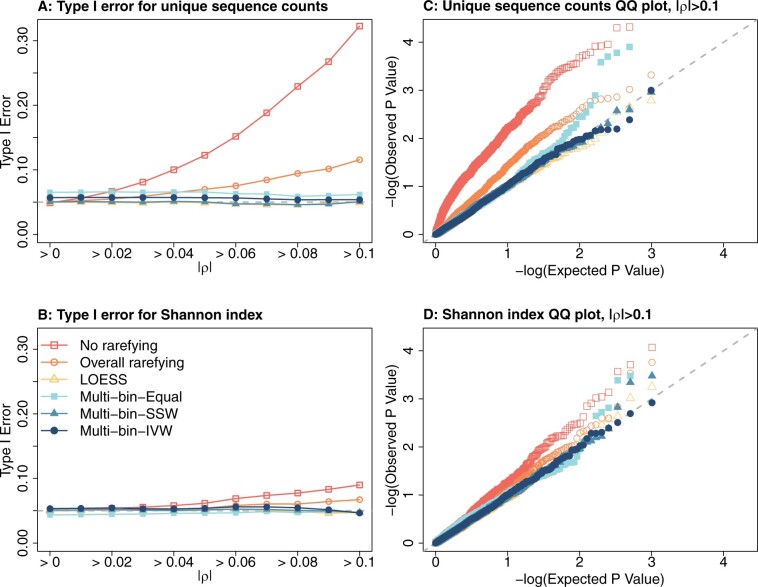
Type I error rates for Simulation A. Panels (A) and (B): type I error rates to all simulated data (|ρ|>0) and restricting to datasets in which |ρ|>0.01,|ρ|>0.02,…,|ρ|>0.1, for the unique sequence counts (A) and Shannon index (B). Panels (C) and (D): QQ plots for the empirical *P*-values and Expected *P*-values (both −log10 transformed), for the unique sequence counts (C) and Shannon index (D).

Figure 1 and Supplementary Fig. S3 summarize the type I error results. Across all the simulated datasets (|ρ|>0), all methods consistently maintained well-controlled type I errors for the unique sequences count (Fig. 1A), the Shannon index (Fig. 1B), bias-corrected Chao1 (Supplementary Fig. S3A), and Pielou’s evenness (Supplementary Fig. S3B). However, with increasing |ρ|, the type I errors in the “No rarefying” and “Overall rarefying” show pronounced inflation in type I errors for the unique sequence counts, bias-corrected Chao1, and Shannon index. The type I error inflation for Pielou’s evenness is less severe, reflecting its resilience to confounding influences from library sizes. Conversely, the multiple versions of the “multi-bin” rarefying approach and the LOESS approach demonstrated well controlled type I errors across all alpha diversity measures, even in scenarios where library size poses a potential confounding factor.

The same conclusions are reached by examining the quantile-quantile (QQ) plots of the empirical *P*-values against the theoretical *P*-values under the null hypothesis, assuming well-calibrated tests (i.e. Uniform distribution between 0 and 1), specifically for datasets where |ρ|>0.1 (Fig. 1C and D, Supplementary Fig. S3C and D). Here, all methods, except for our “multi-bin” approach and the LOESS approach, exhibited significantly inflated type I errors, especially for unique sequence counts, bias-corrected Chao1, and Shannon index. This inflated false positive rate was not as obvious for Pielou’s evenness.

#### 3.1.2 Simulation B

**Figure 2.**
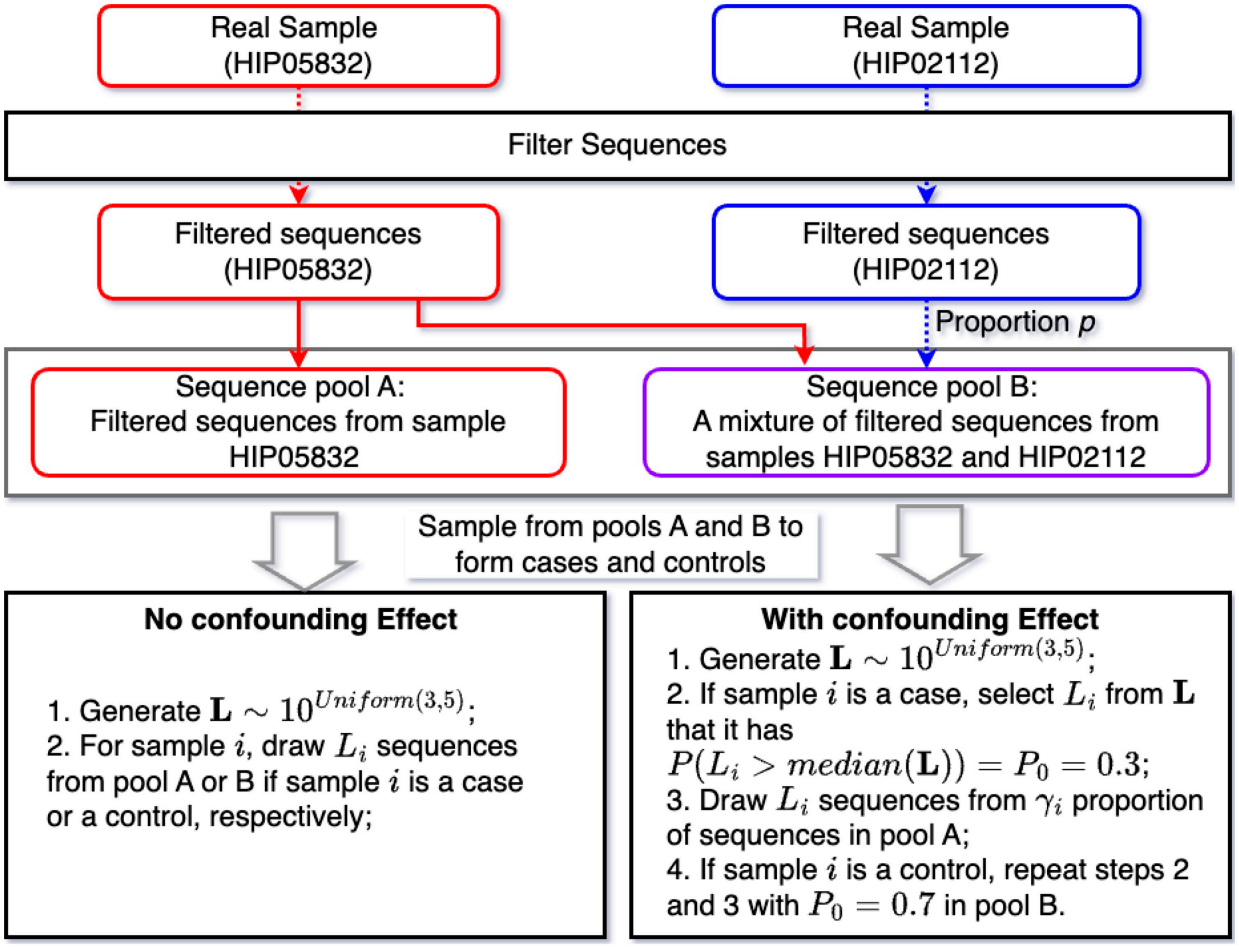
Data generation process for Simulation B.

In Simulation B, using an alternative setup, we designed two distinct scenarios to evaluate the proposed method’s type I error rate and statistical power against alternatives. Data generation involved random sampling with replacement from two unique real samples from the CMV dataset, as illustrated in the flowchart of Fig. 2.

Briefly, samples HIP05832 and HIP02112 were selected as TCR sequence sources, containing 139 764 and 549 611 unique TCR sequences, respectively. These samples exhibited minimal overlap, only 233 shared TCR sequences, resulting in a calculated Bray-Curtis dissimilarity index of 0.9998 (where 1 indicates no shared sequences between two sample TCR profiles). Thus, mixing them would increase the alpha diversity. To ensure sufficient simulation diversity and manage computational complexity, we filtered these samples by removing unique TCR sequences with more than 1000 reads in each sample, which could potentially dominate downstream subsampling. Subsequently, we randomly selected 1000 sequences from each sample.

After filtering, we established two sequence pools: Pool A consisted of all sequences from the filtered sample HIP05832, and Pool B included all sequences from the filtered sample HIP05832 plus a proportion *p* of filtered sample HIP02112. Given the minimal overlap between these samples, Pool B was expected to exhibit greater diversity as *p* increased. Simulations are based on 5000 replications, with each single run simulating a total of 400 samples evenly split between cases (from Pool A) and controls (from Pool B).

We considered two simulation scenarios. In the first simulation scenario, there is no difference in the distribution of library sizes between the case and control groups, such that library size is not a confounding factor. We generated library sizes *L_i_* for each sample *i* from $10^{U}$, where *U* is from a continuous Uniform distribution between 3 and 5. Samples were then constituted by drawing *L_i_* sequences from the corresponding pool, based on its case or control status.

In the second scenario, we deliberately differentiated the library sizes between case and control groups to assess the impact of confounding on alpha diversity. Initially, library sizes for all samples were generated from the same distribution $10^{U}$. We then randomly assign library sizes to the case group using a Bernoulli process so that 70% of the cases had library sizes at or below the median and 30% above, with an inverse distribution for the control group, establishing an intentional correlation between library size and case-control status. To further associate library size with alpha diversity, we ordered all samples by their library sizes in ascending order and divided them into four bins–$M_{1},M_{2},M_{3},M_{4}$–according to set library size intervals [see Equation (1)].

(1) $$\gamma_{i}=\{\begin{array}{cc} 0.8+\frac{0.05(i)}{n_{1}}, & \text{if}\;L_{i}\in M_{1}=[1,000,3,000);\\ 0.95+\frac{0.03(i)}{n_{2}}, & \text{if}\;L_{i}\in M_{2}=[3,000,10,000);\\ 0.98+\frac{0.01(i)}{n_{3}}, & \text{if}\;L_{i}\in M_{3}=[10,000,30,000);\\ 1, & \text{if}\;L_{i}\in M_{4}=[30,000,100,000], \end{array}$$

For each sample *i*, we computed *γ_i_* as per Equation 1, in which (*i*) is the order in library size of sample *i* within the corresponding bin. Here, *γ_i_* denotes the proportion of sequences that can be used from Pool A or B in Step 3 in Fig. 2. Note that our cases were all sampled from Pool A and controls are all sampled from Pool B. For example, if a case group sample *i* falls into *M*_1_ with the second smallest library size ((i)=2), then *γ_i_* is determined by $\gamma_{i}=0.8+0.05\times 2/n_{1}$, where *n*_1_ is the total number of samples that belong to *M*_1_. For the sample with the highest library size in *M*_1_, $\gamma_{i}=0.85$. When $\gamma_{i}=0.85$, we sample from 85% of the sequences from Pool A or Pool B. We conducted this so that the produced *γ*’s for *M*_1_ is linearly even-spaced between 0.8 and 0.85, *γ*’s for *M*_2_ is linearly even-spaced between 0.95 and 0.98, *γ*’s for *M*_3_ is linearly even-spaced between 0.98 and 0.99, *γ*’s for *M*_4_ are all 1. Note that when sampling from the same pool, a larger *γ_i_* implies more sequences included in the sampling space, and the higher diversity we expect, making library size associated with alpha diversity.

**Figure 3.**
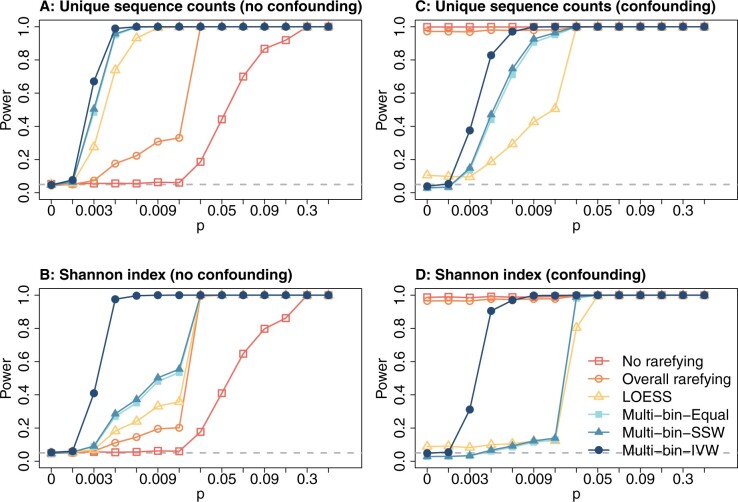
Type I errors and powers in Simulation B. Panels (A) and (B) are under simulations when the library size is not a confounder between alpha diversity and phenotype. Panels (C) and (D) are under simulations when library size confounds the relationship between alpha diversity and phenotype. Note that when *p *=* *0 we are evaluating the type I error.

Figure 3 and Supplementary Fig. S4 shows the type I error (when *p *=* *0) and statistical powers (when *p *>* *0) for all methods across different effect sizes in Simulation B. The left panel (Fig. 3A and B, Supplementary Fig. S4A and B) shows the results when the library size is not a confounding factor between alpha diversity and phenotype. As expected, the powers for all methods increase with higher mixing proportions *p*, and the type I error rates align with the nominal level of 0.05 (grey dashed lines) when *p *=* *0. Our “multi-bin” methods consistently exhibit the highest power for all alpha diversities, outperforming the other three methods.

Among the “multi-bin” approaches, for the unique sequence counts, the Multi-bin-IVW approach shows the highest power, followed by the Multi-bin-SSW and Multi-bin-Equal show similar power. For the Shannon index, Multi-bin-IVW shows the highest power, followed by Multi-bin-SSW and Multi-bin-Equal. Among the competing methods, the LOESS is the most powerful, followed by the “Overall rarefying.” The “No rarefying” method consistently displays the lowest power curve.

In Simulation B when the library size confounds the alpha diversity–phenotype relationship (Fig. 3C and D, Supplementary Fig. S4C and D), it is easy to see that the type I errors at *p *=* *0 are severely inflated for the “No rarefying” and the “Overall rarefying” approaches. It is worth noting that the type I error for simulation B is severely inflated even for Pielou’s evenness, distinct from the earlier simulation scenario. The LOESS method also shows inflated type I error, even though to a lesser extent. The positive correlations between the library sizes, the phenotype labels, and alpha diversities lead to false positive signals of association between alpha diversity and the grouping labels. These findings also challenge the prevailing belief and common practice by demonstrating that overall rarefying is insufficient to adequately address discrepancies in library size across samples.

In contrast, the proposed “multi-bin” approaches consistently maintain well-controlled type I error rates at *p *=* *0. The power of all three “multi-bin” methods for all alpha diversity measures increases as *p* increases. Notably, the Multi-bin-Equal and Multi-bin-SSW methods tend to be slightly conservative. Mirroring the trends observed in simulations without confounding, Multi-bin-IVW emerges as the most potent approach for the richness measures, including unique sequence counts and bias-corrected Chao1, while Multi-bin-SSW and Multi-bin-Equal show comparatively lesser power. For Shannon index and Pielou’s evenness, Multi-bin-IVW exhibits superior power relative to Multi-bin-SSW and Multi-bin-Equal, which again demonstrate comparable power levels.

### 3.2 Analysis of the CMV data

Revisiting the CMV data, the primary objective was to explore the associations between alpha diversities and several clinical covariates, including age, gender, inferred CMV status, and observed CMV status. The dataset of all 666 sample sequence reads is analyzed in this section.

We then applied all the methods in Table 1 to address the potential confounding effect of library sizes across samples in the alpha diversity association analysis. In the “Overall rarefying” approach, $L^{*}=1e6$ was selected as the rarefying level by identifying the saturation “elbow” point in the rarefaction curve in Supplementary Fig. S5A and B. The alpha diversities estimated at each rarefying level (sub-figures C and D of Supplementary Fig. S5) confirmed that $L^{*}=1e6$ was adequate for estimating alpha diversities while retaining the maximum number of samples. All samples with library sizes <1e6 were removed, leaving us with 624 samples for analysis. For the “No rarefying” approach, alpha diversities were directly estimated from the original sequence reads of the samples. For the LOESS method, we fitted a LOESS curve with a smoothing parameter of 0.5 (Aboukhalil and Bulyk 2012).

**Figure 4.**
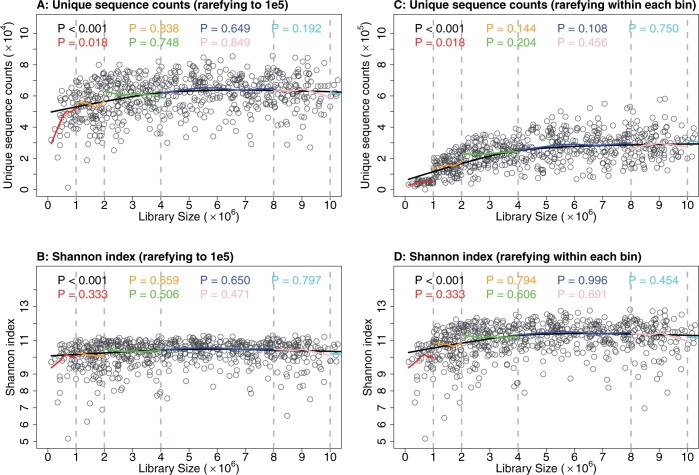
Spearman correlation analyses with scatter plots and LOESS curve fits for alpha diversities within six bins. Panels (A) and (B) analyze unique sequence counts and Shannon index, respectively, against library sizes rarefied to 1e5 across all samples. Panels (C) and (D) follow the same analyses but with samples rarefied to the lowest library size in each bin using the “multi-bin” approach. *P*-values were calculated to test the significance of Spearman correlations between alpha diversities and the library sizes across all samples (black) and within each bin (colored).

We also applied the proposed “muti-bin” approach to the CMV dataset, using five cut points (1e6,2e6,4e6,8e6,1e7) to construct *K *=* *6 bins. The choice of bins was selected by a visual inspection of the relationship between the library size and alpha diversities within each bin (Fig. 4), following the general guidelines in the Materials and Methods section. Notably, across the entire dataset, there is a pronounced association between alpha diversity and library sizes. This holds regardless of whether alpha diversity is estimated by rarefying to the same low level across all samples (Fig. 4A and B) or by rarefying to the lowest level within each specific bin (Fig. 4C and D). When samples are divided into six bins, the association is greatly diminished within each bin. The within-bin associations generally appear nonsignificant, except for the first bin. However, even in this bin, the strength of the association is considerably weaker compared to the combined analysis across all bins. By doing so, we effectively eliminated the confounding effects originating from the library size.

**Figure 5.**
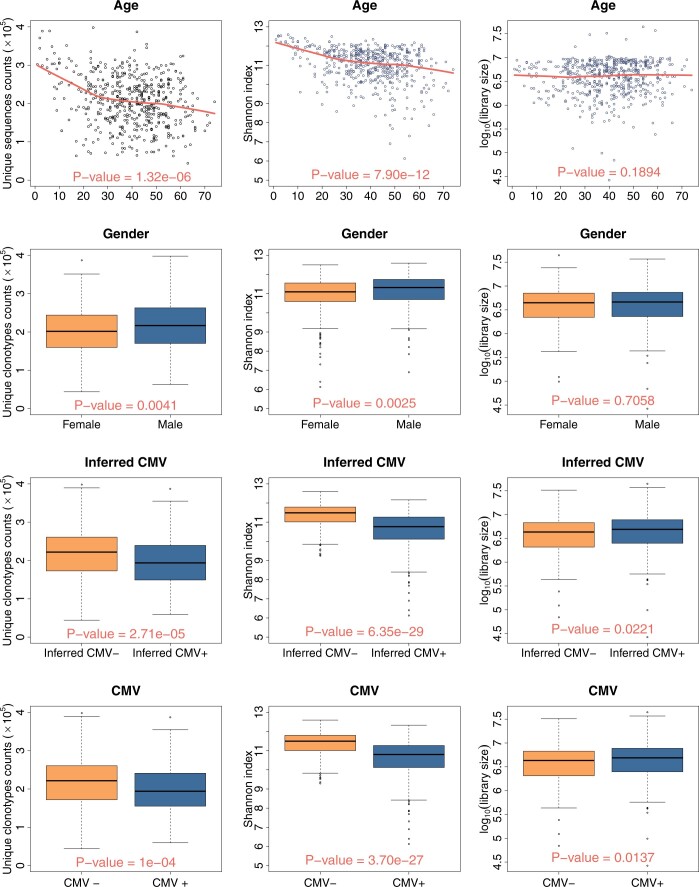
Correlations between covariates and alpha diversity calculated from overall-rarefied samples with $L^{*}=1e6$. (Left column): Plots the unique sequence counts against the of-interested clinical variables. (Middle column): Plots the Shannon index against the of-interested clinical variables. (Right column): Plots the log base 10 transformed library size against the of-interest clinical variables. The curves represent LOWESS fittings for the “Age” variable in the first row. *P*-values at the bottom in each sub-figure are obtained from Spearman rank-based correlation tests. Correlation tests in the right column assess associations between initial library size and clinical variables.

To demonstrate that library size may be a potential confounder in the relationship between alpha diversity and phenotype, we visualized the univariate relationships. This involved plotting the alpha diversity (unique sequences counts and Shannon index) against clinical variables (as shown in the left and middle columns of Fig. 5), and also depicting the relationship between library size and clinical variables (presented in the right column of Fig. 5). Here, all alpha diversities in this analysis were computed using the “Overall rarefying” approach. There are strong associations between both alpha diversities and all clinical variables, including age, gender, and the inferred and observed CMV status (all *P*-values < 0.001). The associations between library size and the clinical variables, though generally weaker, also reveal some significant findings. While there is no significant association between the library sizes and factors such as age and gender (*P*-values > 0.05), significant associations emerge between library sizes and the inferred and observed CMV status. Additionally, the Supplementary Fig. S5E and F shows significant correlations between library size and the alpha diversities by the Spearman rank-based correlation tests. Combined, the “Overall rarefying” method could not fully mitigate the confounding impact of library size on the alpha diversity–phenotype relationship, underlining the necessity of exploring alternative strategies, such as the proposed “multi-bin” approach, to effectively address this issue.

Table 2 and Supplementary Table S2 present the parameter estimations and hypothesis testing outcomes for various methods assessing the association between alpha diversity and key variables. In line with prevailing understanding, library sizes exert a substantial influence on estimated alpha diversities when not adjusted for the “No rarefying” approach. Notably, this association persists as significant even after rarefying all samples to a uniform, lower library size, albeit with a reduced magnitude of association. In this CMV dataset, the LOESS method also successfully removed the alpha diversity-library size relationship.

**Table 2.** Univariate association test results of alpha diversity (unique sequence counts and Shannon index) with various covariates using different normalization methods in the CMV data.^a^

		No. of samples	Age (×1e−4)	Gender (×1e−4)	Inferred CMV (×1e−4)	CMV (×1e−4)	Library size (×1e2)
Unique sequence counts	No rarefying	666	−0.151 (**9.58e−07**)	1.913 (**1.78e−02**)	−1.741 (**3.26e−02**)	−1.238 (1.27e−01)	1.103 (**3.04e−28**)
	Overall rarefying	624	−0.120 (**1.51e−09**)	1.537 (**3.67e−03**)	−2.139 (**5.64e−05**)	−1.950 (**2.27e−04**)	0.273 (**8.66e−05**)
	LOESS	666	−0.155 (**1.29e−10**)	1.877 (**3.09e−03**)	−2.480 (**1.02e−04**)	−2.19 (**5.73e−04**)	0.014 (8.69e−01)
	Bin1	39	−0.059 (**4.53e−03**)	0.703 (1.20e−01)	−0.680 (1.52e−01)	−0.741 (1.35e−01)	1.857 (**3.92e−02**)
	Bin2	104	−0.101 (**6.69e−03**)	1.331 (2.07e−01)	−2.338 (**2.83e−02**)	−2.141 (**4.24e−02**)	2.979 (8.52e−02)
	Bin3	161	−0.081 (**3.85e−02**)	1.868 (7.38e−02)	−3.868 (**1.54e−04**)	−3.687 (**3.00e−04**)	1.425 (1.09e−01)
	Bin4	244	−0.186 (**3.86e−05**)	1.623 (1.85e−01)	−0.514 (6.81e−01)	−0.279 (8.22e−01)	0.948 (7.00e−02)
	Bin5	101	−0.282 (**1.11e−04**)	2.963 (1.21e−01)	−5.739 (**2.54e−03**)	−5.268 (**5.86e−03**)	1.441 (4.36e−01)
	Bin6	14	0.105 (7.23e−01)	1.035 (8.59e−01)	3.346 (5.54e−01)	3.346 (5.54e−01)	−0.211 (3.50e−01)
	Multi-bin-Equal	663	−0.101 (**4.73e−02**)	1.587 (1.32e−01)	−1.632 (1.09e−01)	−1.462 (1.52e−01)	1.407 (**3.18e−03**)
	Multi-bin-SSW	663	−0.149 (**1.51e−10**)	1.775 (**4.55e−03**)	−2.339 (**1.83e−04**)	−2.109 (**7.07e−04**)	1.487 (**2.24e−03**)
	Multi-bin-IVW	663	−0.092 (**2.00e−10**)	1.069 (**2.57e−03**)	−1.472 (**5.43e−05**)	−1.455 (**9.75e−05**)	0.168 (3.72e−01)

		No. of samples	Age	Gender	Inferred CMV	CMV	Library size (×1e7)
Shannon index	No rarefying	666	−0.023 (**5.84e−14**)	0.224 (**4.72e−03**)	−0.791 (**9.94e−25**)	−0.741 (**5.62e−22**)	0.285 (**5.65e−03**)
	Overall rarefying	624	−0.021 (**2.62e−14**)	0.212 (**4.45e−03**)	−0.794 (**1.48e−28**)	−0.758 (**2.68e−26**)	0.018 (8.55e−01)
	LOESS	666	−0.021 (**9.27e−15**)	0.225 (**1.97e−03**)	−0.796 (**8.76e−30**)	−0.762 (**1.61e−27**)	0.079 (4.04e−01)
	Bin1	39	−0.041 (**2.38e−02**)	0.520 (1.69e−01)	−0.729 (6.26e−02)	−0.840 (**3.89e−02**)	7.378 (3.36e−01)
	Bin2	104	−0.023 (2.07e−03)	0.258 (2.14e−01)	−1.060 (**8.59e−08**)	−0.936 (**2.48e−06**)	−2.248 (5.12e−01)
	Bin3	161	−0.017 (**1.86e−03**)	0.186 (2.01e−01)	−0.918 (**8.58e−12**)	−0.937 (**1.99e−12**)	−0.568 (6.47e−01)
	Bin4	244	−0.019 (**3.32e−07**)	0.171 (8.54e−02)	−0.521 (**1.36e−07**)	−0.491 (**5.07e−07**)	−0.015 (9.73e−01)
	Bin5	101	−0.034 (**2.98e−05**)	0.141 (4.99e−01)	−1.030 (**2.01e−07**)	−0.977 (**1.04e−06**)	−1.004 (6.19e−01)
	Bin6	14	−0.014 (7.69e−01)	1.213 (1.63e−01)	−0.970 (2.61e−01)	−0.970 (2.61e−01)	−0.600 (7.24e−02)
	Multi-bin-Equal	663	−0.025 (**3.05e−03**)	0.415 (**9.18e−03**)	−0.871 (**3.89e−08**)	−0.859 (**7.42e−08**)	0.491 (7.34e−01)
	Multi-bin-SSW	663	−0.023 (**1.55e−15**)	0.226 (**2.05e−03**)	−0.801 (**1.66e−32**)	−0.774 (**4.12e−30**)	−0.228 (7.85e−01)
	Multi-bin-IVW	663	−0.021 (**1.46e−16**)	0.201 (**3.96e−03**)	−0.765 (**1.13e−32**)	−0.736 (**1.84e−30**)	−0.423 (8.03e−02)

a Results were derived from univariate linear regression analyses with alpha diversity as the dependent variable. Each cell in the table presents the parameter estimate and its corresponding *P*-value, formatted as “parameter estimate (*P*-value)”. The notation ×1e − 4 at the top of each column indicates that the parameter estimates should be multiplied by 1e − 4 to obtain their true values. Bold *P*-values less than or equal to 0.05 indicate statistical significance in the corresponding association tests.

Upon dividing the samples into multiple bins, our analysis reveals that, apart from the unique sequence counts from Bin 1 (Table 2) and the bias-corrected Chao1 from a few other bins (Supplementary Table S2), there is no significant association between alpha diversities and library sizes within any individual bin. This observation indicates a substantial reduction in the confounding effect of library sizes on the alpha diversity–phenotype relationship. When data are meta-analyzed across all bins, the association between the richness measures (unique sequence counts and bias-corrected Chao1) and library size dramatically decreases. Specifically, the *P*-values from unique sequence counts were recorded at 3.18e−3 for Multi-bin-Equal and 2.24e−3 for Multi-bin-SSW (the similar pattern for the biased-corrected Chao1 in Supplementary Table S2), representing a notable increase from 3.04e−28 observed in nonrarefied data. It is worth noting that the results from “No rarefying” for these two measures from Table 2 and Supplementary Table S2 were identical, as no singletons existed in the dataset for all samples. This pronounced reduction in significance, particularly evident in the case of Multi-bin-IVW with a *P*-value of 0.372 for unique sequence counts (0.081 for bias-corrected Chao1), strongly suggests that the proposed Multi-bin approach effectively mitigates, if not entirely eliminates, the potential confounding influence of library size on the alpha diversity–phenotype relationship. The Shannon index exhibited no association with library sizes, regardless of the weighting approach employed. The Multi-bin-Equal and Multi-bin-SSW approaches indicated no significant association between Pielou’s evenness and library sizes. However, while still significant, the *P*-value for Multi-bin-IVW was reported as 1.02e−02, indicating a decrease from 2.34e−03 observed in nonrarefied data.

The parameter estimates in Table 2 and Supplementary Table S2 were obtained from the simple linear regression model fitting or the LOESS curve fitting under different rarefying methods for the association tests between alpha diversity and clinical variables. Remarkably, all parameter estimates from different rarefying methods consistently show negative associations for age, inferred and observed CMV statuses, and positive associations for the male gender. This suggests that samples from younger individuals, males, and those negative for inferred and observed CMV statuses tend to exhibit higher alpha diversity in sample TCR sequences. For the variables of age and gender without significant associations with total reads, our “multi-bin” methods show approximately the same significant testing *P*-values in Table 2 and Supplementary Table S2 compared to other methods. Regarding the inferred and observed CMV statuses that confounded with sample total reads, all six methods conclude the associations with the alpha diversities are significant. These results further support the robustness and effectiveness of our proposed “multi-bin” approach in handling confounding factors and enhancing the accuracy of the association tests.

### 3.3 TCR sequencing data for nonsmall cell lung cancer

We also re-analyzed the T-cell repertoires from the 571 samples from a cohort of 236 early-stage nonsmall cell lung cancer (NSCLC) patients (Reuben *et al.* 2020). A detailed explanation of the study and data preprocessing can be found in the supplemental file “The NSCLC TCR sequencing study.” Analysis results are presented in Supplementary Table S3.

## 4 Discussion

In this study, we challenge the common practice by demonstrating that simply rarefying all samples to a uniform library size does not adequately mitigate the confounding effect of library sizes on the association between TCR alpha diversity and clinical phenotype. Even after rarefying, the positive correlation between library sizes and alpha diversity measures persists. To address this issue, we introduce a novel “multi-bin” procedure. This method divides the dataset into multiple bins according to sample library sizes, applies rarefying within each bin, and then synthesizes the results across bins using meta-analysis techniques. The “multi-bin” approach effectively diminishes the influence of library size artifacts on alpha diversity results, as evidenced by weaker correlations between alpha diversities and library sizes within each bin. It also ensures no sample (except for sequencing failures) is discarded, minimizing the loss of sequence reads common in overall rarefying methods. Thus, it reaches a balance between robustness and efficiency.

In the “multi-bin” approach, we utilize fixed-effect rather than random-effect meta-analysis, assuming a single, common effect size across all bins due to sampling error alone. This assumption is logical given that library size—a technical artifact—should not inherently influence the alpha diversity–phenotype relationship. However, if there were reasons to suspect distinct relationships in different bins, the random-effect meta-analysis could explore between-bin heterogeneity (DerSimonian and Laird 1986, Han and Eskin 2011). Options within the random-effect meta-analysis framework include the classical random-effects meta-analysis technique (DerSimonian and Laird 1986), incorporating between-bin heterogeneity into the null hypothesis, or the modified version from Han and Eskin (2011), which typically provides greater statistical power by not assuming heterogeneity under the null.

We compared three weighting methods for our “Multi-bin” approach in simulation studies and real data analysis. The inverse variance weighting strategy exhibited the highest statistical power and is the preferred choice. However, it is essential to recognize that the inverse variance strategy relies on the assumption of homogeneity between individual bins. In cases where homogeneity cannot be assumed, the sample size weighting strategy provides a more conservative conclusion with less risk of false positives, as it assigns more weight to larger sample size bins. This alternative helps mitigate the impact of heterogeneity and produces more stable and reliable results. Equal weighting usually generates the lowest power and is not recommended.

The selection of cutting thresholds is crucial for our approach, impacting data partitioning and analysis reliability. These thresholds aim to ensure minimal variation in alpha diversity within each bin due to library sizes. However, statistical significance is contingent upon both sample size and effect size. Consequently, in instances with a smaller sample size, multiple threshold configurations may fulfill the stipulated criteria. Conversely, in cases featuring larger sample sizes and a narrow window of library sizes, it may be challenging to identify cut points that meet all predefined conditions. Moreover, differences in sequencing methods (e.g. bulk versus single-cell) and the specific TCR chains sequenced (e.g. alpha chain or beta chain or both) can result in diverse levels and patterns of diversity. These disparities may complicate the selection of appropriate thresholds for binning. Our multi-bin procedure was motivated by the analysis of the bulk TCR sequencing experiments. However, the same approach should also be applicable to single-cell TCR sequencing. Our simulation studies show that even with suboptimal threshold selection, our approach effectively reduces inflated Type I errors compared to traditional methods. The findings underscore the resilience of the “multi-bin” approach in mitigating analytical biases associated with sequencing depth and library size discrepancies, making it a preferable alternative for abundance datasets with uneven sequencing efforts from ecology and microbial diversity studies.

## Supplementary Material

btae431_Supplementary_Data

## Data Availability

All immunosequencing data underlying this study are freely available and can be downloaded and analyzed from the immuneACCESS sites at https://doi.org/10.21417/B7001Z and https://doi.org/10.21417/AR2019NC.
