# Supplementary material for: A multi-bin rarefying method for evaluating alpha diversities in TCR sequencing data
Source: Bioinformatics. 2024 Jul 1;40(7):btae431. doi: 10.1093/bioinformatics/btae431 (PMC11246167; doi:10.1093/bioinformatics/btae431)
Supplement: btae431_Supplementary_Data [file btae431_supplementary_data.pdf]

A multi-bin rarefying method for evaluating alpha diversities in TCR sequencing data

Supplementary Materials

Supplementary Tables

Table S1: Summary of patient characteristics of the CMV data used for real data analysis and simulations

	$n = 666$	SD/Percentages
Age		
Mean	39.54	14.03
Gender		
Males	297	44.6%
Females	345	51.8%
Unknown	24	3.6%
Inferred CMV status		
Inferred CMV+	364	54.7%
Inferred CMV -	275	41.3%
Unknown	27	4.1%
Virus Diseases		
Cytomegalovirus+	352	52.9%
Cytomegalovirus-	289	43.4%
Unknown	25	3.8%

SD: the standard deviation.

Table S2: Univariate Association Test Results of Alpha Diversity (Chao1 and Pielou's evenness) with Various Covariates Using Different Normalization Methods in the CMV Data

		# of Samples	Age (×1e−4)	Gender (×1e−4)	Inferred CMV (×1e−4)	CMV (×1e−4)	Library size (×1e2)
Bias-corrected Chao1	No rarefying	666	-0.151 (**9.58e-07**)	1.913 (**1.78e-02**)	-1.741 (**3.26e-02**)	-1.238 (1.27e-01)	1.103 (**3.04e-28**)
	Overall rarefying	624	-0.149 (**2.65e-08**)	2.058 (**3.79e-03**)	-2.123 (**3.03e-03**)	-1.896 (**7.77e-03**)	0.535 (**7.62e-09**)
	LOESS	666	-0.155 (**1.29e-10**)	1.877 (**3.09e-03**)	-2.480 (**1.02e-04**)	-2.190 (**5.73e-04**)	0.014 (8.69e-01)
	Bin1	39	-0.168 (**2.14e-03**)	1.752 (1.48e-01)	-0.966 (4.51e-01)	-0.951 (4.78e-01)	8.116 (**3.82e-04**)
	Bin2	104	-0.106 (**1.14e-02**)	1.463 (2.07e-01)	-2.421 (**3.88e-02**)	-2.252 (5.20e-02)	4.525 (**1.65e-02**)
	Bin3	161	-0.084 (**4.23e-02**)	2.012 (6.69e-02)	-3.913 (**2.78e-04**)	-3.662 (**6.55e-04**)	2.161 (**2.01e-02**)
	Bin4	244	-0.189 (**5.39e-05**)	1.637 (1.96e-01)	-0.400 (7.57e-01)	-0.167 (8.96e-01)	1.253 (**2.00e-02**)
	Bin5	101	-0.283 (**1.12e-04**)	2.970 (1.20e-01)	-5.754 (**2.52e-03**)	-5.278 (**5.88e-03**)	1.509 (4.15e-01)
	Bin6	14	0.104 (7.39e-01)	2.080 (7.35e-01)	3.390 (5.71e-01)	3.390 (5.71e-01)	-0.118 (6.25e-01)
	Multi-bin-Equal	663	-0.121 (**2.59e-02**)	1.986 (7.61e-02)	-1.677 (1.24e-01)	-1.486 (1.73e-01)	2.908 (**6.89e-07**)
	Multi-bin-SSW	663	-0.157 (**8.41e-11**)	1.920 (**3.14e-03**)	-2.339 (**3.27e-04**)	-2.092 (**1.26e-03**)	2.401 (**3.45e-06**)
	Multi-bin-IVW	663	-0.143 (**6.90e-12**)	1.837 (**9.37e-04**)	-2.429 (**1.40e-05**)	-2.236 (**6.97e-05**)	0.360 (8.14e-02)
		# of Samples	Age (×1e2)	Gender (×1e2)	Inferred CMV (×1e2)	CMV (×1e2)	Library size (×1e8)
Pielou's evenness	No rarefying	666	-0.140 (**1.05e-14**)	1.310 (**6.18e-03**)	-5.948 (**3.21e-39**)	-5.767 (**3.42e-37**)	-0.188 (**2.34e-03**)
	Overall rarefying	624	-0.133 (**1.90e-13**)	1.211 (**1.25e-02**)	-5.803 (**1.25e-36**)	-5.599 (**2.03e-34**)	-0.098 (1.23e-01)
	LOESS	666	-0.128 (**3.16e-13**)	1.271 (**6.03e-03**)	-5.691 (**2.96e-38**)	-5.518 (**2.71e-36**)	0.076 (2.05e-01)
	Bin1	39	-0.270 (**1.32e-02**)	2.742 (2.35e-01)	-6.198 (**7.58e-03**)	-7.053 (**3.28e-03**)	3.409 (4.66e-01)
	Bin2	104	-0.140 (**7.29e-03**)	1.380 (3.43e-01)	-8.018 (**4.15e-09**)	-7.112 (**2.20e-07**)	-3.240 (1.75e-01)
	Bin3	161	-0.121 (**7.47e-04**)	0.974 (3.06e-01)	-6.137 (**2.62e-12**)	-6.357 (**2.19e-13**)	-1.052 (1.86e-01)
	Bin4	244	-0.102 (**4.91e-06**)	1.031 (8.17e-02)	-4.085 (**1.48e-12**)	-3.971 (**3.09e-12**)	-0.283 (2.72e-01)
	Bin5	101	-0.202 (**1.57e-04**)	0.520 (6.98e-01)	-6.795 (**7.35e-08**)	-6.491 (**3.54e-07**)	-1.249 (3.34e-01)
	Bin6	14	-0.150 (6.49e-01)	9.881 (1.06e-01)	-8.906 (1.40e-01)	-8.906 (1.40e-01)	-0.421 (7.85e-02)
	Multi-bin-Equal	663	-0.164 (**4.40e-03**)	2.755 (**1.06e-02**)	-6.690 (**2.09e-10**)	-6.648 (**3.17e-10**)	-0.473 (6.01e-01)
	Multi-bin-SSW	663	-0.139 (**6.34e-14**)	1.281 (**6.91e-03**)	-5.839 (**2.02e-43**)	-5.712 (**2.62e-41**)	-0.867 (1.12e-01)
	Multi-bin-IVW	663	-0.124 (**1.65e-14**)	1.113 (**1.08e-02**)	-5.359 (**1.25e-42**)	-5.234 (**3.25e-41**)	-0.415 (**1.02e-02**)

Note: Results were derived from univariate linear regression analyses with alpha diversity as the dependent variable. Each cell in the table presents the parameter estimate and its corresponding p-value, formatted as "parameter estimate (p-value)". The notation ×1e−4 at the top of each column indicates that the parameter estimates should be multiplied by 1e−4 to obtain their true values.

The Non-Small Cell Lung Cancer (NSCLC) TCR sequencing study

We re-analyzed the TCR Sequencing data from the 571 samples from a cohort of 236 early-stage NSCLC patients (Reuben et al., 2020). The samples were collected from peripheral blood mononuclear cells (PBMCs), uninvolved tumor-adjacent lungs, or tumors. Data were downloaded from https://doi.org/10.21417/AR2019NC. For quality control, we removed the samples from other body locations (i.e., Lymph node tissue) and samples with library sizes less than 1e4. We also dropped the sequences containing "*" or "NA" in their sequence reads and the sequences that don't start with cysteine (C) and end with phenylalanine (F). Finally, the filtered data set contains 478 samples from 219 patients. On average, 12,478 ($\pm 102,356$ s.d.) unique TCR β sequences are observed on each sample, and the sequencing depth across samples ranged from 10,088 to 251,706.

All the methods in Table 1 (main manuscript) were applied to the NSCLC data set to normalize the TCR sequencing data. We fitted univariate linear regression models to assess the association between the normalized alpha diversity (unique sequence counts, bias-corrected Chao1, Shannon index, and Pielou's evenness) and both (the original unrarefied) library size and tissue type. We set up contrasts to assess the tissue type effect (PBMC VS. Normal, PBMC VS. Tumor, and Tumor VS. Normal). For the "Overall rarefying" approach, we selected $L^* = 1e4$ as the rarefying level. For the LOESS method, the smoothing parameter was set as 0.5. In our "multi-bin" approaches, we constructed five bins using four cut points at 2e4, 3e4, 4e4, and 5e4. All other settings were the same as the analysis setup for the CMV dataset outlined in the main text.

Table S3 presents the parameter estimations and hypothesis testing outcomes for this analysis. Library sizes affect the estimation of alpha diversities in the "No rarefying" approach. Even with "Overall rarefying", the associations between alpha diversities and library size remain significant. The LOESS method and our multi-bin approaches can effectively mitigate the confounding effects of library size. In each specific bin, alpha diversity shows no association with library sizes across all combinations of bins and alpha diversity measures, except for the Shannon Index and Pielou's evenness in Bin 5. Even in cases where there are significant associations, the strength of the association is quite weak. This indicates a successful mitigation of the confounding effects of library sizes. For the proposed Multi-bin approaches, after meta-analysis, all measures of alpha diversity cease to show any association with library size, with the sole exception of Pielou's evenness when using the Multi-bin-IVW method, which only shows a borderline significance.

Comparing the different tissue types, our Multi-bin approaches reveal significant differences in alpha diversity across various tissue types. Specifically, PBMC samples exhibit considerably higher diversity compared to both Normal and Tumor tissues when measured by unique sequence counts, the Shannon Index, and Bias-corrected Chao 1. However, when assessing Pielou's evenness, only the Multi-bin-IVW method identifies a notable difference between PBMC and Normal samples. Additionally, the Multi-bin-IVW approach indicates a significantly higher alpha diversity in Tumor samples compared to Normal samples across unique sequence counts, the Shannon Index, and Pielou's evenness, but not for Bias-corrected Chao 1. Other Multi-bin methods do not detect any differences in alpha diversity measures between Tumor and Normal samples. These findings align with the conclusions drawn by Reuben et al. (2020).

Table S3: Univariate Association Test Results of Alpha Diversity with the Library Size and Tissue Types Using Different Normalization Methods in the NSCLC Data

		# of Sample	PBMC VS. Normal (×1e−4)	PBMC VS. Tumor (×1e−4)	Tumor VS. Normal (×1e−4)	Library size
Unique sequence counts	No rarefying	478	-3.677 (**1.17e-83**)	-3.559 (**1.67e-81**)	-0.118 (3.88e-01)	0.501 (**8.03e-164**)
	Overall rarefying	478	-0.493 (**9.55e-156**)	-0.437 (**4.55e-139**)	-0.056 (**3.84e-07**)	0.035 (**1.92e-40**)
	LOESS	478	-0.671 (**2.38e-12**)	-0.799 (**6.24e-17**)	0.127 (1.25e-01)	0.00 (9.79e-01)
	Bin1	214	-0.571 (**5.97e-74**)	-0.546 (**2.89e-70**)	-0.024 (**3.52e-04**)	0.023 (3.85e-01)
	Bin2	112	-0.964 (**1.47e-51**)	-0.918 (**1.12e-50**)	-0.046 (**4.66e-02**)	0.144 (2.10e-01)
	Bin3	45	-1.382 (**1.63e-12**)	-1.315 (**1.13e-14**)	-0.068 (5.68e-01)	0.197 (5.66e-01)
	Bin4	32	-2.162 (**1.02e-06**)	-2.00 (**4.18e-11**)	-0.162 (6.70e-01)	0.670 (2.99e-01)
	Bin5	75	-1.906 (**1.01e-02**)	-1.873 (**4.07e-08**)	-0.033 (9.66e-01)	-0.010 (6.20e-01)
	Multi-bin-Equal	478	-1.397 (**1.06e-17**)	-1.33 (**6.12e-68**)	-0.067 (7.01e-01)	0.205 (1.60e-01)
	Multi-bin-SSW	478	-1.055 (**1.86e-19**)	-1.011 (**9.98e-84**)	-0.044 (7.23e-01)	0.106 (8.17e-02)
	Multi-bin-IVW	478	-0.686 (**0.00e+00**)	-0.675 (**0.00e+00**)	-0.026 (**4.37e-05**)	0.006 (7.25e-01)
		# of Sample	PBMC VS. Normal	PBMC VS. Tumor	Tumor VS. Normal	Library size (×1e5)
Shannon index	No rarefying	478	-2.121 (**3.71e-80**)	-1.782 (**7.52e-64**)	-0.339 (**3.72e-05**)	1.719 (**2.09e-37**)
	Overall rarefying	478	-1.123 (**1.53e-41**)	-0.840 (**1.90e-26**)	-0.282 (**3.00e-05**)	0.646 (**1.38e-11**)
	LOESS	478	-0.550 (**2.33e-09**)	-0.463 (**3.07e-07**)	-0.087 (2.78e-01)	-0.119 (2.34e-01)
	Bin1	214	-1.724 (**1.51e-19**)	-1.579 (**7.04e-17**)	-0.145 (**1.24e-02**)	1.231 (3.24e-01)
	Bin2	112	-1.369 (**7.00e-18**)	-1.122 (**3.59e-14**)	-0.247 (**5.72e-03**)	3.079 (1.38e-01)
	Bin3	45	-1.068 (**1.53e-02**)	-0.896 (**1.21e-02**)	-0.172 (6.32e-01)	5.740 (2.77e-01)
	Bin4	32	-1.675 (**1.26e-02**)	-1.352 (**6.17e-04**)	-0.323 (6.36e-01)	4.149 (5.23e-01)
	Bin5	75	-0.532 (6.65e-01)	-0.199 (7.01e-01)	-0.333 (8.00e-01)	-0.559 (**3.34e-02**)
	Multi-bin-Equal	478	-1.274 (1.20e-05)	-1.03 (4.70e-12)	-0.244 (4.22e-01)	2.728 (1.13e-01)
	Multi-bin-SSW	478	-1.389 (**1.51e-10**)	-1.176 (**1.11e-21**)	-0.213 (3.24e-01)	2.003 (**4.21e-02**)
	Multi-bin-IVW	478	-1.475 (**5.35e-49**)	-1.224 (**5.26e-39**)	-0.177 (**1.99e-04**)	-0.412 (9.98e-02)
		# of Sample	PBMC VS. Normal (×1e−5)	PBMC VS. Tumor (×1e−5)	Tumor VS. Normal (×1e−5)	Library size
Bias-corrected Chao1	No rarefying	478	-1.789 (**1.47e-85**)	-1.773 (**4.38e-86**)	-0.016 (8.09e-01)	2.179 (**1.77e-105**)
	Overall rarefying	478	-0.733 (**2.28e-114**)	-0.722 (**3.81e-114**)	-0.011 (6.07e-01)	0.588 (**6.09e-42**)
	LOESS	478	-0.423 (**7.79e-12**)	-0.515 (**7.21e-17**)	0.092 (8.64e-02)	0.038 (5.86e-01)
	Bin1	214	-0.494 (**9.01e-108**)	-0.49 (**1.93e-106**)	-0.004 (2.61e-01)	-0.267 (2.13e-01)
	Bin2	112	-0.621 (**4.06e-55**)	-0.615 (**7.34e-56**)	-0.006 (6.46e-01)	0.54 (4.70e-01)
	Bin3	45	-0.972 (**2.28e-11**)	-0.963 (**5.63e-14**)	-0.009 (9.22e-01)	0.904 (7.20e-01)
	Bin4	32	-1.376 (**8.73e-05**)	-1.352 (**7.96e-09**)	-0.024 (9.40e-01)	5.427 (2.33e-01)
	Bin5	75	-1.574 (**3.31e-02**)	-1.588 (**1.91e-06**)	0.014 (9.86e-01)	0.247 (1.88e-01)
	Multi-bin-Equal	478	-1.008 (**2.08e-10**)	-1.002 (**1.08e-43**)	-0.006 (9.71e-01)	1.37 (1.85e-01)
	Multi-bin-SSW	478	-0.797 (**6.53e-12**)	-0.794 (**1.31e-55**)	-0.004 (9.76e-01)	0.494 (2.51e-01)
	Multi-bin-IVW	478	-0.529 (**0.00e+00**)	-0.53 (**0.00e+00**)	-0.004 (2.25e-01)	0.051 (7.10e-01)
		# of Sample	PBMC VS. Normal (×1e2)	PBMC VS. Tumor (×1e2)	Tumor VS. Normal (×1e2)	Library size (×1e7)
Pielou's evenness	No rarefying	478	1.378 (**4.85e-02**)	2.176 (**1.65e-03**)	-0.798 (1.99e-01)	-5.053 (**5.47e-12**)
	Overall rarefying	478	-0.602 (3.36e-01)	0.557 (3.68e-01)	-1.159 (**3.80e-02**)	-1.77 (**8.05e-03**)
	LOESS	478	-0.465 (4.79e-01)	0.553 (3.93e-01)	-1.018 (8.18e-02)	-0.49 (4.84e-01)
	Bin1	214	-5.569 (**1.30e-04**)	-4.99 (**6.35e-04**)	-0.579 (2.27e-01)	-9.653 (2.73e-01)
	Bin2	112	-1.714 (8.91e-02)	-0.106 (9.13e-01)	-1.607 (**1.66e-02**)	1.432 (9.01e-01)
	Bin3	45	1.573 (6.53e-01)	2.2 (4.38e-01)	-0.627 (8.31e-01)	39.413 (3.24e-01)
	Bin4	32	-2.053 (6.82e-01)	-0.973 (7.28e-01)	-1.081 (8.40e-01)	4.443 (9.11e-01)
	Bin5	75	3.603 (7.18e-01)	7.065 (9.70e-02)	-3.463 (7.46e-01)	-5.561 (**1.00e-02**)
	Multi-bin-Equal	478	-0.832 (7.24e-01)	0.639 (5.96e-01)	-1.471 (5.49e-01)	6.015 (6.03e-01)
	Multi-bin-SSW	478	-2.319 (1.89e-01)	-1.008 (3.15e-01)	-1.31 (4.54e-01)	-0.851 (8.98e-01)
	Multi-bin-IVW	478	-2.684 (**6.21e-04**)	-1.063 (1.48e-01)	-0.93 (**1.50e-02**)	-5.418 (**6.97e-03**)

Note: Results were derived from univariate linear regression analyses with alpha diversity as the dependent variable. Each cell in the table presents the parameter estimate and its corresponding p-value, formatted as "parameter estimate (p-value)". The notation ×1e−4 at the top of each column indicates that the parameter estimates should be multiplied by 1e4 to obtain their true values.

Supplementary Figures

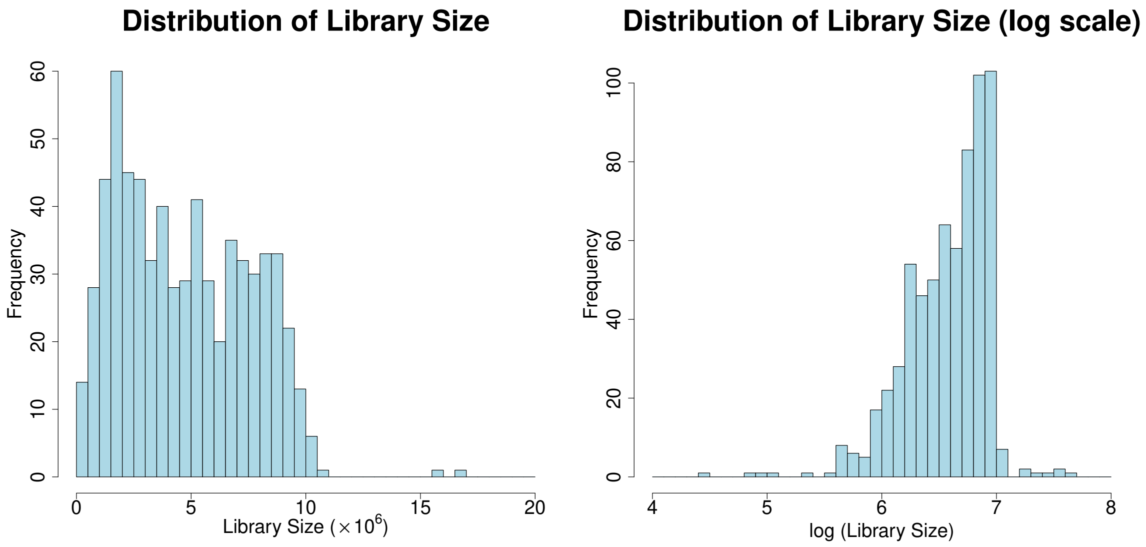

Figure S1: The distributions of the sequencing depth/library size from 666 samples from the CMV study.

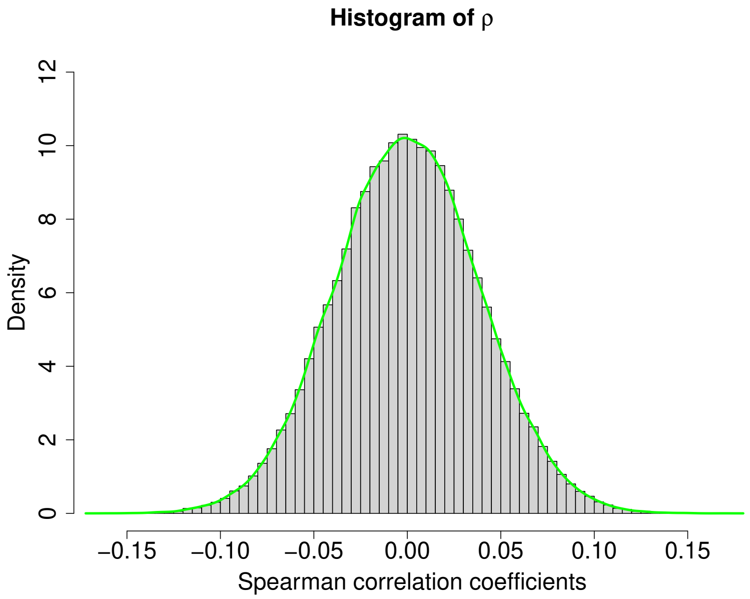

Figure S2: Histogram of the estimated Spearman correlation coefficients (ρ) between library size and simulated binary phenotype across 100,000 simulations.

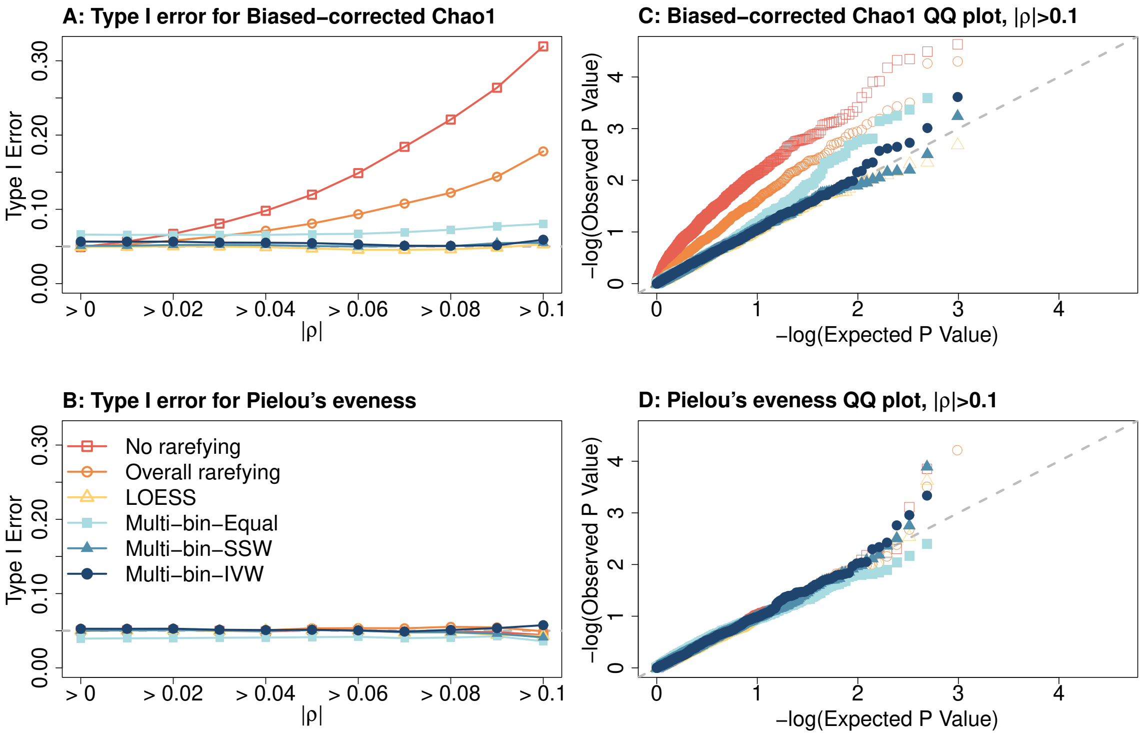

Figure S3: Type I error rates for Simulation A. Panel **A** and **B**: type I error rates to all simulated data ($|\rho| > 0$) and restricting to datasets in which $|\rho| > 0.01, |\rho| > 0.02, \ldots, |\rho| > 0.1$, for bias-corrected Chao1 (**A**), and Pielou's evenness (**B**). Panel **C** and **D**: QQ plots for the empirical p-values and Expected p-values (both -log10 transformed), for bias-corrected Chao1 (**C**) and Pielou's evenness (**D**).

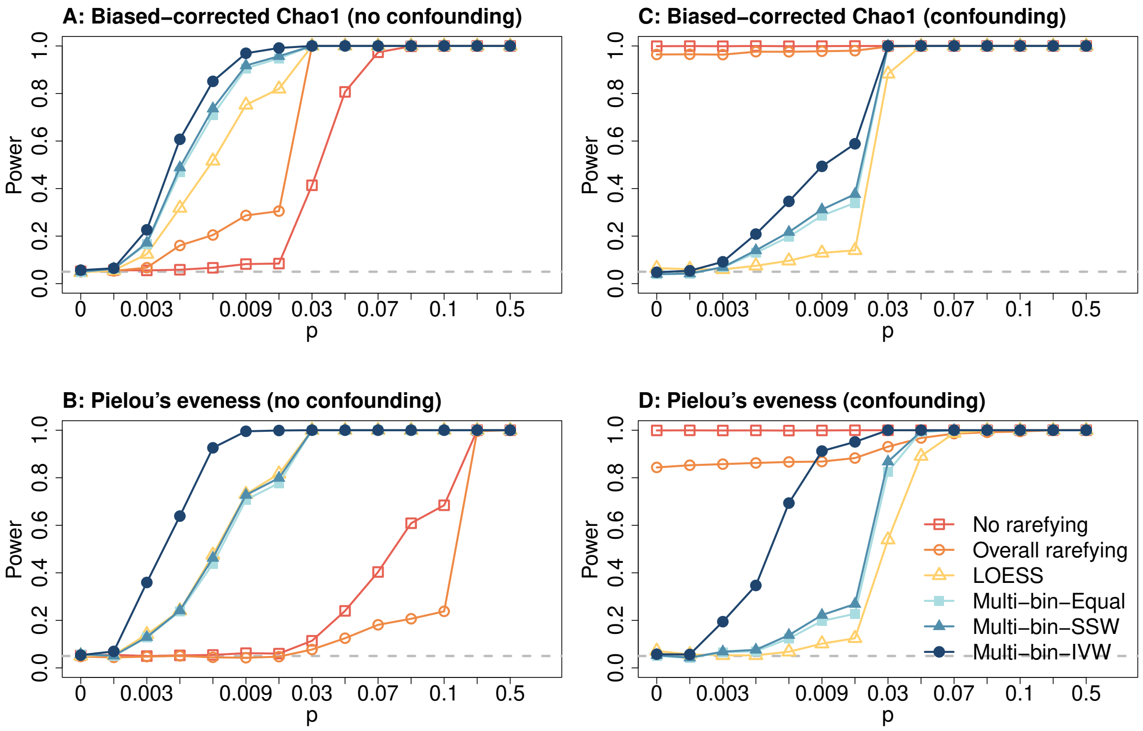

Figure S4: Type I errors and powers in Simulation B. Panel **A** and **B** are under simulations when the library size is not a confounder between alpha diversity and phenotype. Panel **C** and **D** are under simulations when library size confounds the relationship between alpha diversity and phenotype. Note that when $p = 0$ we are evaluating the type I error.

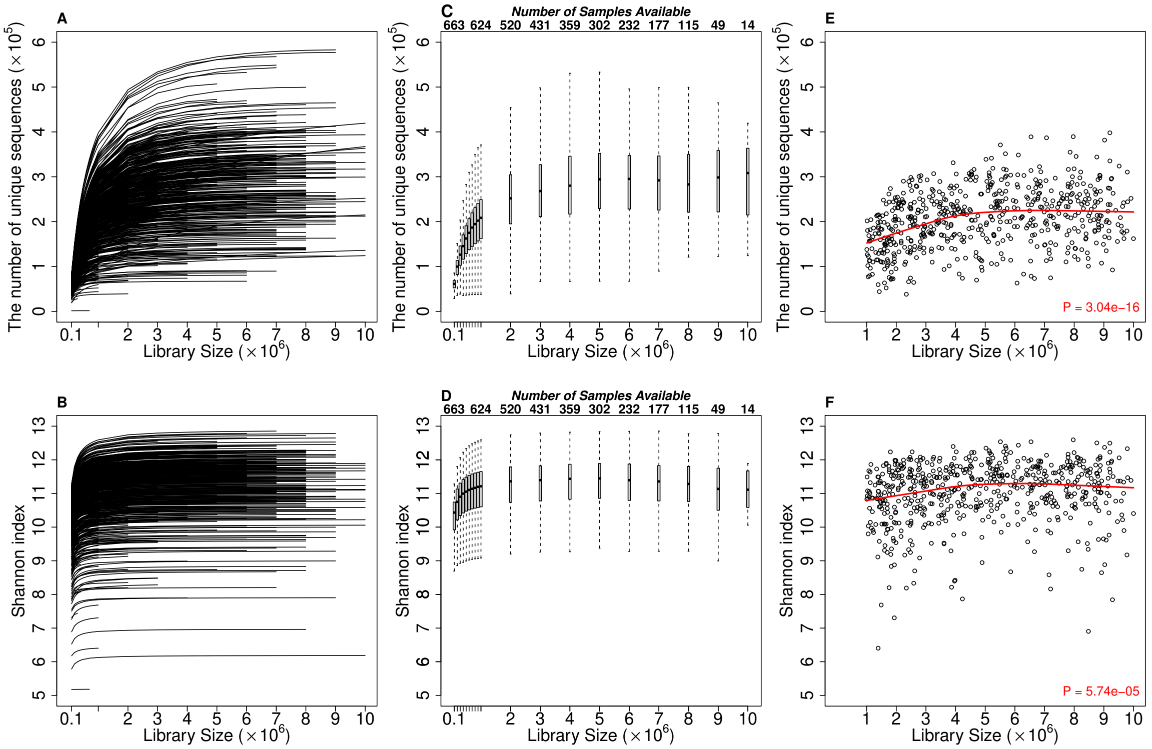

Figure S5: Rarefaction curves for the CMV dataset **(A)**: Rarefaction curves for the unique sequence counts. **(B)**: Rarefaction curves for Shannon index. **(C)**: Boxplots of the unique sequence counts at multiple rarefying levels. **(D)**: Boxplots of Shannon index at multiple rarefying levels. **(E)**: A scatter plot of the unique sequence counts (calculated by rarefying all samples to 1e6) vs the original library sizes. **(F)**: A scatter plot of the Shannon index (calculated by rarefying all samples to 1e6) vs the original library sizes. For **(E)** and **(F)**, the LOESS curves are marked in red. The p-values at the bottom are obtained from the Spearman correlation test between the library size and the alpha diversity.

References

Reuben, A., Zhang, J., Chiou, S.-H., Gittelman, R. M., Li, J., Lee, W.-C., Fujimoto, J., Behrens, C., Liu, X., Wang, F., et al. (2020). Comprehensive t cell repertoire characterization of non-small cell lung cancer. *Nature communications*, *11*(1), 603.
